# Cohort Profile: A Scottish population-level study on the outcomes of people released from prison (RELEASE)

**DOI:** 10.1093/ije/dyag220

**Published:** 2026-10-21

**Authors:** Catriona Connell, Richard Kjellgren, Kate Hunt, Jan Savinc

**Affiliations:** Salvation Army Centre for Addiction Services and Research, School of Public Health and Society, University of Stirling, Stirling, United Kingdom; Salvation Army Centre for Addiction Services and Research, School of Public Health and Society, University of Stirling, Stirling, United Kingdom; Institute for Social Marketing and Health, School of Public Health and Society, University of Stirling, Stirling, United Kingdom; School of Health and Social Care, Edinburgh Napier University, Edinburgh, United Kingdom; Scottish Centre for Administrative Data Research, Edinburgh, United Kingdom

**Keywords:** public health, prison, mental health, substance use, administrative data, resettlement

Key Features

- RELEASE is the first population-level cohort study of health outcomes among people released from prison in the UK and is among the first internationally to use a matched comparison group without recent prison exposure.
- RELEASE was established to advance policy and practice, to improve the health outcomes of people released from prison, and to improve wider public health. It now includes a series of linked studies.
- RELEASE uses national Scottish linked administrative prison, health, and death records. Using routinely recorded administrative data avoids the limitations of longitudinal studies that rely on primary data collection with this population.
- The RELEASE cohort contains almost all people released from Scottish prisons in 2015 (index year) and a demographically matched (ratio 1:5), random general-population sample who had not been in prison.
- The RELEASE cohort includes 49 526 people aged 16–87 years (mean 34.8 years). Data are available from 1 January 2010 to 28 February 2020, allowing ≤5 years of follow-up and the examination of pre- and post-release variables.
- RELEASE data include demographic details, health services records (community prescriptions; outpatient, inpatient, unscheduled, and emergency), prison services (admissions, releases), and national death registry records.
- For information about RELEASE, contact catriona.connell@stir.ac.uk.

## Why was the cohort set up?

The RELEASE cohort was established to compare the health outcomes of people who had experienced imprisonment in Scotland, with a demographically matched general-population sample who had not experienced imprisonment. The RELEASE study was originally funded by the Chief Scientist Office, Scotland (CSO; HIPS/21/54). RELEASE is now a program of studies. Funders include the CSO (HIPS/25/19) and the National Institute for Health and Care Research (NIHR504345). People with imprisonment experience continue to be involved as co-investigators and through Lived Experience Advisory Panels. Additional involvement and advisory panels are set up on a per-study basis from parties spanning policy, practice, and academia.

Release into the community is inevitable for almost all people in prison. The number of people released annually likely exceeds 30 million people internationally [1]. Health inequalities in the prison population are well documented [2]. International literature consistently reports insufficient transitional/post-release support and that dealing with competing priorities (e.g. securing accommodation, finances) in the context of structural barriers (e.g. inaccessible services, disadvantaged communities) post-release can reduce access to healthcare and contribute to poor health and increased mortality [3–8].

Comprehensive understanding of post-release healthcare access (and wider outcomes) is limited by a lack of large longitudinal cohort studies. Such studies are challenging to achieve by using primary data collection with this population, as sampling/recruitment bias and sample attrition can impact the reliability and validity of conclusions. Research using linked administrative data is increasingly pursued to address these limitations, particularly in Australia [9, 10] and North America [11, 12]. However, many of the cohorts lack a well-matched comparison group who have not been imprisoned. To develop evidence-informed services and support for people released from prison (hereafter “people released”), there is a need for more studies using linked administrative data to examine varied health conditions and multi-morbidity; to examine a range of services; and to compare outcomes to robustly matched comparators, particularly in countries with different health, social, and legal contexts.

Using national data from Scotland, RELEASE is the first population-level research using linked administrative prison, mortality, and health data to examine post-release outcomes in the UK.

Scotland is impacted by some of the worst and increasing health inequalities, and has the second-largest per-capita prison population in Western Europe [13, 14]. Like many nations, Scotland’s prison population is predominantly male, on average younger than the general population, and more likely to be from socio-economically deprived areas [15], reflecting structural inequities that disproportionately impact young men [16]. People from ethnic minorities are not overrepresented in the Scottish prison population. The prison population is rising in Scotland, similarly to those in many nations, with overcrowding impacting healthcare and rehabilitative programming [17]. With more people being released with unmet health needs, into a context of inequalities and strained public services, it is critical to know how services can be optimized to improve individual and public health outcomes.

Though part of the UK, justice, health, and social-care policies are devolved to the Scottish Government. Healthcare is universally available and free at point of use (from the National Health Service, funded through general taxation). When the RELEASE cohort was being established (details below), 13 of Scotland’s 15 prisons were publicly run by the Scottish Prison Service (SPS) and two were privately run but are now in the SPS estate. Most hold a mixed-sentenced and unconvicted (“remand”) population. One prison each holds women, young people, men on long sentences, and men in open conditions (with greater freedom to leave the prison for rehabilitative activity). Release from different establishments can be identified in the data.

The RELEASE data allow research on any health condition across health services ranging from primary to emergency care and permits robust comparisons between those who have and have not been in prison. The RELEASE cohort includes everyone released from prison over 1 year and a demographically matched general-population sample (important due to the demographically skewed prison-population profile). It contains multiple health and criminal justice variables, to control for potential confounding and compare outcomes. RELEASE data therefore permit comparison to a matched general-population sample, published general-population data, and comparison based on demographic, health-related, and criminal-justice-related factors.

The first research using the RELEASE cohort examined contact with health services that was related to mental health and substance use, comparing people with and without prison experience [18] and examining variation in rates by demographic, health-related, and criminal-justice-related factors [19]. Mental health and substance use were of primary concern owing to the high rates of diagnoses of/problems with mental health and substance use among the prison population [2]; the starkly elevated rates of avoidable mortality among those released, in which substance use and suicide are leading causes [20]; and the public health impact of high emergency-service use among people released, which is largely driven by emergencies for substance use and mental health [21, 22]. These problems are acute in Scotland, where suicide and alcohol-related death rates exceed those of other UK nations and drug-related death rates are amongst the highest internationally [16].

## Who is in the cohort?

### Study design

RELEASE is an observational cohort study in which national population-level linked administrative data from prison, health, and deaths records are used.

The RELEASE cohort consists of two groups. The first is all people released from Scottish prisons in 2015 (exposed group, *n* = 8313). The second is a matched then randomly selected general-population sample who had not been imprisoned between 2010 and 2015 (unexposed group, *n* = 41 213). Matching was at a ratio of 1:5 on age, sex, postcode district/area (see below), and a postcode-derived indicator of deprivation [Scottish Index of Multiple Deprivation (SIMD)] [23], and who were alive on the date their respective exposed individual was released. The total cohort includes 49 526 people.

Preparatory work to create the cohort began in 2020. Due to disruption during/following the COVID-19 outbreak, the release year was set as 2015 to allow ≤5 years of follow-up at a time of stable service availability, so that research findings could inform contemporary service (re)design. Service and contextual changes since 2015 must be considered, depending on the topic under review, e.g. changes in available treatment [24]. However, the demographic profile of the Scottish prison population remains largely unchanged. There is a slight increase in average age (due to reduced imprisonment of children/young people and greater prosecution for historical sexual offenses) and a slight increase in ethnic diversity (but prison levels are below national levels) [25].

### Methods to create the cohort

An independent third party—the Electronic Data Research and Innovation Service (eDRIS) at Public Health Scotland (PHS, a national public health organization) facilitated the creation, linkage, and de-identification of the cohort for approved researchers to access in a secure environment.

The SPS provided data on demographics, prison admissions, and releases (2010–20) from their “PR2” operational management system. Using probabilistic matching (on name, date of birth, address), 8491 of 8917 people released (95.2%) were successfully matched to their community health index (CHI) number in the CHI database (population spine for healthcare purposes). A CHI number is allocated at birth, at a person’s first health service contact, or at death. The CHI database is the most comprehensive record of the Scottish population, though it does not record migration. Someone who immigrated to Scotland, was imprisoned, but did not use health services would be missing from the CHI database (no linkage would be achieved). Somebody who emigrated post-release would have no post-release health records. These cases are rare, as is evident from the 95.2% linkage rate.

To add the unexposed groups, five matched individuals per person released who (i) had not been in prison in the preceding 5 years (determined from PR2) and (ii) were alive on the date their respective exposed individual was released were selected from the CHI database. This was done in stages, with matching rules relaxed stepwise where numbers of matched individuals were low. Individuals were randomly selected at each stage at which there were more than five available matches.

Demographic details were extracted from the CHI database (age, sex assigned at birth, postcode, and SIMD). Additional variables were extracted from PR2 for those released (ethnicity, disclosed disability, religious affiliation, sexual orientation, marital status, veteran status, transgender status). Ethnicity is (historically) poorly reported in the CHI database and was thus extracted only from PR2 for the exposed group. However, the exposed group was 96% White, consistently with the national census results for the time [26].

Cohort prison and health records were linked (2010–20). PR2 records include demographics, admission dates, release dates, and the circumstances of release. Health datasets include community prescribing (PIS), outpatient (SMR00), community drug and alcohol (SDMD), general inpatient (SMR01), psychiatric inpatient (SMR04), unscheduled care [24-hour telephone support (NHS24), out-of-hours general practice (OOH-GP)], and emergency [Scottish Ambulance Service (SAS), accident and emergency department (A&E)] service use. Death records were linked from 2015 to 2020.Once linked, data were deposited in a secure research environment. Approved researchers accessed the data to clean, check linkage accuracy, spot check for errors, and finalize the RELEASE cohort. Individuals erroneously included in the matched unexposed group (due to initial use of an incorrect reference dataset) and their matched counterparts were removed. The linkage process achieved a well-matched cohort, described in the first RELEASE results paper [18], allowing robust between-group comparisons.

## How often have they been followed up?

The data allow follow-up without attrition (excluding where individuals have died) over any defined period up to 28 February 2020. This date was chosen as it was immediately prior to the onset of the COVID-19 “lockdown” in Scotland (see above). Work is planned to extend follow-up and expand the range of outcomes.

The first RELEASE study examined contact with health services for mental health and substance use for the 4 years following release in 2015 and the same period for matched unexposed counterparts, up to 31 December 2019 [18].

## What has been measured?

The RELEASE data include selected variables from each of the linked health, deaths, and prison datasets (datasets listed above, described in the RELEASE protocol) [27], which allows the examination of healthcare contacts for any health condition, mortality, and imprisonment episodes over a 10-year period. Variables differ between datasets and full variable specification is available from the RELEASE team. Additional variables facilitate statistical control for multiple covariates, including socio-demographic characteristics, prior health service contact, comorbidities, and re-imprisonment. Mortality records allow censoring for deaths.

The outcome in the initial RELEASE study was the rate of contact with each service recorded as related to mental health, substance use, or “dual diagnosis” (both mental health and substance use recorded in a contact). Owing to dataset variation, contacts attributable to these conditions were operationalized differently between datasets. For example, inpatient datasets (SMR01, SMR04) categorized diagnosis using the International Classification of Diseases 10th Edition (ICD-10), whilst the outpatient dataset (SMR00) has a poor recording of diagnosis and thus the recorded clinical speciality was used (e.g. general psychiatry). This is described in the RELEASE protocol paper, with coding available on GitHub [27, 28].

Demographic data for all cohort members include age, sex, postcode-derived urban/rurality indices, and SIMD. Postcode is not available, but the health-board area in which someone resides/contacts a service is recorded. Additional demographic variables extracted from PR2 for the exposed group include sexual orientation, religion, marital status, disclosed disability, veteran status, and transgender status. These rely on self-report on admission to prison and it is possible that some people may be unwilling to disclose such information. PR2 variables include the prison that someone is released from and the type of release (e.g. on “license” or “from court”).

Projects are underway examining mortality (HIPS/25/19), healthcare contacts, and re-imprisonment (NIHR504345). Analysis plans will be available on the project’s Open Science Foundation (OSF) repository [29].

## What has it found?

The RELEASE team have examined contact related to mental health and substance use with all services over 4 years post-release, calculating adjusted incidence-rate ratios [aIRRs: adjusted for pre-index mental health, substance use, or dual diagnosis contacts; pre- and post-index time in community (accounting for re-imprisonment and death); and comorbidities] to compare rates of contact between exposed and unexposed groups with each service and for each of mental health, substance use, and dual diagnosis [18].

Results show elevated rates of contact related to mental health and substance use across all services in the exposed group compared with the unexposed group. This is despite robust matching, censoring for deaths, and controlling for multiple covariates. For full results, see the first RELEASE manuscript [18]. Notably, the aIRRs between the exposed and unexposed groups tended to be larger for substance use than for mental health, particularly for community prescriptions [substance use (SU) = 5.95 (4.83, 7.32), metal health (MH) = 1.80 (1.67,1.94)], psychiatric admissions [SU = 10.74 (6.12, 18.84), MH = 3.62 (2.39, 5.49)], and emergency-department presentations [SU = 7.98 (5.71, 11.17), MH = 4.88 (3.78, 6.29)]. Differences in contact rates between exposed and unexposed groups increased as services progressed in the acuity of conditions addressed/urgency of need, particularly for mental health [community prescriptions = 1.80 (1.67, 1.94), outpatient = 2.61 (2.17, 3.16), psychiatric admissions = 3.62 (2.39, 5.49), ambulance calls = 7.75 (5.76, 10.42)].

One in four people released had at least one ambulance-service contact for mental health or substance use in the 4 years post-release (7.8 times the rate of the unexposed group for mental health, 7.6 times for substance use). One in five people released had an emergency-department contact for mental health or substance use in the 4-year follow-up (4.9 times the rate in the unexposed group for mental health, 8.0 for substance use). It is vital to explore the reasons for this, to ensure that timely, appropriate healthcare is accessible to people released from prison.

Demographic, health-related, and criminal-justice-related factors associated with increased rates of contact related to mental health and substance use contacts were examined in the exposed group [19].

## What are the main strengths and weaknesses?

RELEASE is the most comprehensive UK research program to date for understanding and improving post-release outcomes. The exposed group is a national cohort of all people released and the unexposed group was matched on key variables then randomly selected from the whole population. RELEASE has a large sample size (nearly 50 000) and contains care and prison trajectories spanning 10 years. RELEASE cohort creation used high-quality data-linkage procedures. Data for the cohort include healthcare records from community to emergency care, death, and prison records. It provides the basis for epidemiological analyses of multiple outcomes and service contacts, monitoring trends, and informing evidence-based policy and practice developments that are critical to improving individual and public health outcomes.

RELEASE involved linking national routine administrative data from Scotland. This may limit generalizability (e.g. to countries with different systems, services, and demographic profiles). Though advantageous compared with primary data collection, research using administrative data is constrained by which variables are routinely collected and, for health data, who presents to services. There is potential for recording/coding practice to vary over time/place and some variables were self-reported. PR2 data were reconstructed into prison episodes, which relied on assumptions in which there were inconsistencies between records (details in protocol) [27]. The RELEASE dataset does not yet include full primary care data, but contains community prescriptions as a partial proxy.

## Can I get hold of the data? Where can I find out more?

The RELEASE dataset is held in the National Safe Haven, hosted by PHS, and cannot be shared beyond the RELEASE team at present. However, as data custodians, the RELEASE team welcomes contact from researchers. Analysis plans are available on the RELEASE OSF page [29], code on GitHub [28], and publications on the RELEASE website (www.release.stir.ac.uk). Please direct enquiries to catriona.connell@stir.ac.uk.

## Ethics approval

This study was approved by the University of Stirling General University Ethics Panel (7144) and the Public Benefit and Privacy Panel for Health and Social Care (2021–0145). It conforms to the standards required to conduct health research in Scotland’s National Safe Haven.

## Data Availability

The data underlying this article cannot be shared due to data protection restrictions. The RELEASE data were extracted and linked following approval by the Public Benefit and Privacy Panel, Health & Social Care (PBPP-HSC). The data are stored in a secure trusted research environment and access is restricted to the project team. We welcome contact from researchers.
